# Effects of Walking Speed on Total and Regional Body Fat in Healthy Postmenopausal Women

**DOI:** 10.3390/nu14030627

**Published:** 2022-01-31

**Authors:** Jacquelyn M. La New, Katarina T. Borer

**Affiliations:** School of Kinesiology, The University of Michigan, Ann Arbor, MI 48109, USA; jqq@umich.edu

**Keywords:** speed of walking, total fat loss, abdominal fat loss, lean body mass

## Abstract

Introduction: This study had two aims: (1) To confirm the efficacy of exercise speed and impulse (session duration at a given speed) to produce total and abdominal fat loss in postmenopausal women, and (2) compare the exercise speed and impulse necessary for the stimulation of fat loss to the suppression of bone mineral loss. Of special interest was to compare these parameters of exercise on fat loss in the same study and with the same subjects where they were found to suppress bone mineral loss. We hypothesized that (1) more total fat will be lost with slow walking and a longer impulse than with fast speed and shorter impulse, and (2) more abdominal subcutaneous (SC) and visceral fat (VF) will be lost with fast walking speed. Materials and Methods: Fat loss and suppression of bone mineral loss were measured in the same 25 subjects after 15 weeks, and fat measurements were also taken after 30 weeks in 16 residual subjects. Study parameters were walking a 4.8 km distance 4 days/week at either 6.6 km/h (120% of ventilatory threshold (VT)) or at 5.5 km/h (101.6% of VT) and expending 300 kcal/session. Body composition (fat and lean body mass, LBM) was measured with dual-energy X-ray absorptiometry (DXA) and anthropometric methods. Results: Slow walkers in the residual group progressively lost a significant percent of total body fat over 30 weeks while no such loss occurred after 15 weeks in fast walkers in either group, supporting hypothesis 1. However, the 20% higher starting body fat in 16 residual slow relative to fast subjects suggests that exercise fat loss is greater in overweight than in lean subjects. In fast walkers, fat loss occurred after 30 weeks of training. Hypothesis 2 was not supported as both speeds led to equal VF loss in 30-week group as estimated by waist circumference (CF) confirming that VF responds to the magnitude of energy expenditure and not the walking speed. Conclusions: Total body fat is lost through walking at all speeds, but the change is more rapid, clear, and initially greater with slow walking in overweight subjects. A longer exercise impulse at a lower speed in our study initially produced greater total fat loss than a shorter one with fast walking speed. This was reversed in comparison to how the same exercise in the same subjects suppressed bone mineral loss. Data from other studies indicate that longer impulses may promote greater fat loss at both slow and high exercise speeds, and our study providing only a 4.8 km walking distance may have limited the walking impulse and the magnitude of fat loss. Increased exercise energy expenditure at either walking speed produces equivalent declines in visceral fat in postmenopausal women, and with sufficiently long impulses, should reduce disabilities associated with central obesity.

## 1. Introduction

The rising level of obesity in the United States includes the post-menopausal population. Between 1960 and 2020, obesity in women and men in the U.S. increased more than threefold, from 13.4 to 41.9% [1,2]. Overweight, defined as body mass index (BMI) of between 25 and 30 kg/m^2^, and obesity, as BMI above 30 kg/m^2^, often leads to pathologies such as diabetes, atherosclerotic vascular disease that may precipitate stroke, heart attack, pulmonary thromboembolism, respiratory failure, and cancer [3,4,5]. Mortality risk rises in parallel with increases in obesity [6,7,8,9]. In addition to the usual general causes of obesity, principally due to low levels of physical activity [10] and overeating [11], menopause and its associated decline in circulating estradiol contribute to female weight gain and redistribution of body fat from predominantly subcutaneous, gluteal, and femoral locations, the so-called gynoid body fat pattern, to an android pattern featuring greater deposits of abdominal subcutaneous (SC) and visceral fat (VF) [12,13,14,15]. The android pattern of fat distribution has a much stronger association with metabolic complications of obesity such as hypertension, diabetes, coronary heart disease, and blood hypercoagulability, and increased risk of death [16,17,18,19], making successful fat loss for postmenopausal women important. Understanding whether walking speed can facilitate fat loss in postmenopausal women represents the first compelling reason for this study. The second compelling reason is that the group of subjects used in the present study has already demonstrated that the higher walking speed, accompanied by a 45-min-long daily exercise session or impulse, was effective in suppressing bone mineral loss and moderately increasing leg and whole-body bone mineral density [20]. Two subsequent studies have confirmed this finding and added the additional requirements for the anabolic responses of bone to exercise: A pre-exercise meal [21] and the necessity for a 40- to 45-min, but not shorter, impulse or duration of exercise session [22]. Different features of exercise appear to be necessary to produce specific physiological effects. To increase insulin sensitivity, the duration of exercise sessions (170 min vs. 115 min) was found to be necessary and more effective than exercise intensity (75% vs. 45% of VO_2_ peak) [23]. It was, therefore, of interest to explore whether a difference in exercise speed (over 6 km/min in fast walkers vs. 5.5 km/min in slow walkers) and session duration (45 min in fast walkers vs. 59 min in slow walkers), that was differentially affecting bone mineral loss in healthy postmenopausal women, would also differentially affect total and regional fat loss.

Meta-analyses [24] and reviews [25,26] attest that the efficacy of exercise in producing total and regional fat loss is not clearly understood. Unsettled issues include questions regarding why total exercise-induced body fat loss seldom exceeds 3 kg [26] or 2 to 3% of initial body weight [25] regardless of the duration of exercise training, and whether there is a clear dose–response relationship between frequency, volume, and intensity of exercise on one hand, and total body fat loss on the other. Any claim that larger doses of exercise produce proportional increases in total fat loss is weakened by reports that a higher weekly frequency (5 days/week vs. 3 days/week) of daily 60-min moderate-intensity exercise, generating weekly exercise energy expenditures (EEEs) of 1047 and 1709 Kcals, respectively, produces an almost indistinguishable fat loss of −1.1 and −1.3 kg [27]. It has also been reported that over a 10-month duration, expenditure of 400 or 600 kcal per session produced no significant difference in the magnitude of fat loss [28]. The role of exercise intensity or walking speed on the magnitude of total body fat loss has similarly revealed contradictory results. Studies either showed no difference in fat loss if the exercise was isocaloric at different intensities [29,30,31] or indicated that fat loss was inversely proportional to exercise speeds. In a study that involved the same distance of 4.8 km/day to be walked at three different speeds, 8 km/h, 6 km/h, and 4 km/h, the magnitude of fat loss was inversely related to walking speed, that is, the greatest amount of total body fat loss occurred at 4 km/h [32]. Some others reported greater fat loss at higher walking speed or intensity [33,34] and others at a lower speed or intensity [35,36]. 

Information about different effects of exercise on SC abdominal and VF depots also needs to be re-examined because of the regional redistribution after menopause of SC fat from the peripheral gynoid to abdominal android SC and VF sites [12,13,14,15]. The redistribution of fat to the abdominal region after menopause carries serious health risks for postmenopausal women [16,17,18,19]. Seven studies focused on different features of exercise that affect abdominal SC and VF. One study [37] demonstrated that over a period of one year, an 1800 daily step increment above 7000 walking steps significantly decreased SC and VF by 20.1 and 22.7%, respectively. Six studies indicated that abdominal SC fat declines more than VF with higher walking speed, intensity, or weekly frequency, while VF declines in response to negative energy balance regardless of how it is achieved [38,39]. In a previous study [38], one group of subjects expended 28,776 kcal after 20 weeks of steady endurance training and another group expended 13,838 kcal during 15 weeks of high-intensity intermittent training (HIIT). Despite about two-fold difference in the exercise energy expenditure (EEE) of the two training paradigms, SC fat loss after HIIT was 2.3% greater in trunk sites and 5.2% greater in limb sites compared to that after aerobic training. When energy expenditure at two different exercise frequencies per week (1.5 days/week vs. 3.5 days/week) was made isocaloric by adding a 240-kcal dietary deficit to the low-frequency group, SC fat loss was inversely proportional to exercise frequency and was about 5 to 5.5 times greater in the highest compared to the lowest exercise frequency. By contrast, VF loss was proportional to the negative energy balance achieved by either dieting plus exercise or exercise alone and not related to exercise frequency [39]. In subjects exercising for 16 weeks at either a low intensity below the VT or at a higher intensity above the VT, abdominal fat did not change after the low-intensity exercise but declined significantly after the high-intensity exercise [33]. Another study [40] reported that longer session duration (and associated EEE) of exercise per week at the same moderate intensity produced twice as much VF loss with 195 min/week than with 135 min/week. Two studies from the same research group [41,42] demonstrated that high exercise intensity produces greater SC fat loss than lower intensity, but when combined with a longer session bout, SC fat loss at high intensity was amplified. The study design pitted two weekly distances, a short distance of 17.6 km (SD) and a 32-km long distance (LD), against two exercise intensities, 48% and 78% of VO_2_ peak. In the first study [41], comparing four skinfolds (SFs) and four circumferences (Circ) between the SD groups differing in exercise intensity, the higher intensity led to a 40% greater SF loss and 20% greater Circ loss. However, when the higher intensity was coupled with the greater LD distance, SC fat loss was 2.4 to 2.5 times greater than in the SD-low intensity group. In the second study [42], computed tomography scans were used to directly assess the magnitude of both abdominal SC fat and VF. The study outcome was similar. Higher-intensity SD exercise led to 2.6 times greater SC fat loss and 32% greater VF loss than SD exercise at a low intensity. However, when the high intensity was coupled with a greater-volume LD exercise, SC fat loss was 19.2 times greater and VF loss was 11.5 times greater than in the SD low-intensity group. Finally, in an isocaloric study where exercise was performed at 80 and 59% of VO_2_ peak, no difference in the fat loss in the sum of seven skinfolds and seven circumferences was reported for the two intensities [31].

In view of the contradictory data published regarding the direction of the change and magnitude of total and regional fat loss, we felt justified to posit two hypotheses. The first one was prompted by the evidence suggesting that lower exercise intensity facilitates body fat loss, while higher walking speed suppresses it [32,35,36]. This hypothesis is also supported by data that exercise intensity affects the rate of body lipid utilization [43]. During exercise lasting more than one hour at a relative effort of between 25% and 45%, about 90% of EEE is supplied by free fatty acids (FFA) and fat oxidation, but the rate of energy utilization is low. As exercise intensity and walking speed increase above 36% of relative effort and above the ventilatory threshold (VT), EEE is increasingly supported by carbohydrates and anaerobic metabolism and therefore is less likely to produce fat loss.

**Hypothesis** **1.** *Lower-speed and longer duration of aerobic exercise over a 4.8 km distance will produce greater total body fat loss than isocaloric exercise at a higher speed.*

Our second hypothesis is supported by suggestive evidence of greater SC fat loss at higher walking speeds and relative intensities [33,38,39,41,42].

**Hypothesis** **2.** *Higher exercise speed will produce greater abdominal SC fat loss than isocaloric exercise at a lower speed.*

## 2. Materials and Methods

### 2.1. Subjects

Forty-two healthy sedentary postmenopausal women were recruited from the Ann Arbor area over a five-year period using the web page UMClinicalStudies.com (accessed on 18 December 1997 and 18 December 1998) and newspaper advertisements to participate in an early-morning mall-walking program. The subjects in the present study were the same 25 subjects who exercised 15 weeks for measurements of bone mineral loss reported previously [20] and fat measurements presented in this study. In the present study, 16 residual subjects continued exercising for another 15 weeks for a total of 30 weeks. Study inclusion criteria were 50–70 years old, surgical or natural menopause with no menstrual periods for at least one year, no metabolic disease, a body mass index (BMI) of less than 35 kg/m^2^, non-smoker, the absence of musculoskeletal disabilities that would prevent mall walking, and sedentary status (<60 min of regular exercise per week). Exclusion criteria included the presence of hormonally uncorrected hypothyroidism and any deviations from inclusion criteria. Training started for each subject upon enrolment. Twenty-five subjects completed 15 weeks of training after 17 subjects dropped out. One subject was excluded from the study for pre-menopausal status and one for participating in a dieting program, four for not adhering to DXA measurement requirements, and eleven women left the study due to inconvenience or lack of time, relocation to another city, and other personal reasons. Sixteen subjects completed 30 weeks of training after a loss of another nine participants for some of the same reasons as during the first 15 weeks of the study. The study was conducted in accordance with the Declaration of Helsinki, and the protocols HUM1997-521, 18 December 1997–12 November 1998 and HUM1998-0335, 18 December 1998–December 2003) were approved by the University of Michigan Medical School Institutional Review Board (IRB-MED). All subjects signed informed consent for human studies approved by the IRB-MED. Baseline subject characteristics did not differ between the 15-week (*n* = 25) and 30-week (*n* = 16) groups (Table 1). 

Baseline characteristics for the subgroups categorized by speed assignment are in Table 2. Age, years post menopause, weight, BMI, VO_2_ max, VT, and relative efforts at VT did not differ between slow and fast walkers in 15-week and 30-week groups. The only exception was percent body fat, which was significantly lower in the fast- than in the slow-walking 30-week group, and the difference in body fat mass between the fast and slow walkers approached significance.

### 2.2. General Experimental Protocol

All subjects received health screenings from their own healthcare providers, completed a health history questionnaire, and underwent body composition assessments and treadmill fitness tests. Measurements of height, weight, and total body fat by a dual-energy X-ray absorptiometry (DXA) apparatus (model Prodigy, Lunar Radiation Corporation, Madison, WI, USA) were performed at the Michigan Clinical Research Center (MCRU) and the Ann Arbor Veteran’s Administration Hospital. The same skilled technician performed the anthropometric measurements of regional fat distribution at the University of Michigan Exercise Endocrinology Laboratory. Subjects were matched by age, weight, BMI, and VT after a determination of their VT. They were assigned to two different walking speeds, below or above their VTs. Participants received information about the purpose of the study, which included walking under supervision at a local mall at assigned speeds and a premeasured 4.8 km distance five days a week for 15 or 30 weeks. The exercise was isocaloric as the assigned 4.8 km distance was the same for slow and fast walkers, and the speed of locomotion over the same distance does not affect the amount of energy expended [44]. Walking started either at 6:30 when the mall opened or at 8:00. New subject cohorts began morning mall walking at the beginning of academic semesters. Women received reminder cards with individually assigned speeds and target times to achieve for each 0.8 km mall landmark. Starting at 1 km/day, the daily distance increased by one km each week and reached the target distance by week 4. As walkers passed each mile marker, walking supervisors provided encouragement and called out time elapsed to assist walkers in maintaining assigned speeds. Subjects prevented from walking with the group were asked to mall-walk at another time on the same day or on a weekend day and to record their own times. Subjects were asked not to alter their customary physical activities or dietary practices while participating in the study. However, to determine whether any changes in body weight could be due to changes in food intake, subjects completed three-day food diaries at the study baseline and after 15 and 30 weeks of walking.

### 2.3. Determination of Ventilatory Threshold and Speed Assignment

VT was used to assign two speeds as it represents a physiological marker of a switch from the metabolism relying on the predominant oxidation of lipids as exercise metabolic fuel to an increasing dependence on anaerobic carbohydrate utilization [43,45]. An exercise intensity of 65% of relative effort and VT mark the point of equality between lipid and carbohydrate fuel use. Above the VT, when the ventilatory rate increases above the rate of oxygen consumption, the rate of total caloric expenditure progressively rises and allows a shift in fuel metabolism toward greater, and ultimately predominant, carbohydrate utilization. This shift is due to a circulatory restraint preventing high FFA concentrations at high exercise intensities from reaching exercising muscles [43]. VT assessment [45,46] entailed walking on a level treadmill starting at 2.4 km/h with subjects breathing through a mouthpiece and tubing into a Sensor-Medic metabolic cart (Yorba Linda, CA, USA). Speed was raised at three-minute intervals in increments of 0.6 km/h until reaching 3 km/h after which it increased by 0.3 km/h. This continued until subjects felt compelled to break into a run or when the respiratory quotient (RQ, the ratio between VCO_2_ and VO_2_) increased above 1.0, the indicator of maximal aerobic capacity. VT was determined as the intersection between two regression lines, one representing parallel rates of VE and VO_2_ at lower exercise intensities and the other representing hyperventilation generated at a rate higher than VO_2_ at high intensities. Low-speed assignment in the SLOW group (*n* = 15) was to exercise at intensities below 95% of VT, and high-speed assignment in the FAST group (*n* = 11) concerned intensities above 125% of VT. 

### 2.4. Body Composition

Dual-energy X-ray absorptiometry (DXA) and anthropometric body composition were measured at baseline and after 15 weeks and 30 weeks of training. A DXA scanner at MCRU (Prodigy, Lunar Radiation Corporation, Madison, WI, USA, with DXL software version 1.3y, pencil beam mode) was used to assess the mass and percent of total fat and lean tissue of the whole body, arms, and legs. Anthropometric methods for the assessment of subcutaneous fat entailed measurements of skinfolds (SKFs) and circumferences (Circs) at select body parts. SKFs were measured at five sites with Lange SKF calipers (Creative Health products, Knoxville, TN, USA). The measurements, to the nearest 0.5 mm, were taken in triplicate using specified procedures [47,48] at the abdomen, supra-iliac, mid-thigh, triceps, and subscapular sites. Circs estimated changes in body fat as well as in lean body mass and were measured in centimeters to the nearest mm by the same experienced technician who also took skinfold measurements. The seven Circs were abdomen at the narrowest waist (abdomen 1) and at the umbilicus (abdomen 2), upper arm extended, forearm, hips at the gluteal site, thigh closest to the trunk, and calf. Anthropometric data were also used to estimate percent body fat using equations described by Teran [49] and Jackson [50] that included both Circs and SKFs and utilized the Siri equation [51] after determining body density [50]. The Teran Equation [49] utilizes the natural logarithm of the sum of Circs of the upper arm, forearm, abdomen 1 and abdomen 2, thigh and calf, the sum of triceps and thigh SKFs, the forearm Circ, as well as residual lung volume defined as (0.016 × age) + (0.01) − (8 × height) − 2.261. The Teran equation is 43.427 × natural log(upper arm + abdomen1 + abdomen2 + thigh + calf Circs) + 10.906 × natural log(triceps + thigh SKFs) − 0.694 forearm Circ − 4.045 residual lung volume − 236.298. We used two Jackson [50] equations. Equation (7) includes the sum of 4 SKFs and age: 1.096095 − 0.0006952 × (triceps + abdomen + suprailium + thigh SKFs) + 0.0000011 × (triceps + abdomen + suprailium + thigh SKFs)^2^ − 0.0000714 × age). Jackson Equation (11) also includes the gluteal Circ: = 1.1454464 − 0.0006558 × (triceps + abdomen + suprailium + thigh SKFs) + 0.0000015 × (triceps + abdomen + suprailium + thigh SKFs)^2^ − 0.0000604 × age − 0.0005981 × gluteal Circ.

### 2.5. Meals

Subjects took their meals at home. To assess the quality and quantity of food consumed, subjects completed food diaries at baseline, 15 weeks, and 30 weeks. All food eaten was recorded for three consecutive days consisting of at least one weekday and one weekend day. The total nutrient intake and type of macronutrients consumed recorded in the three-day diaries were analyzed using the Nutritionist III computer program (N-Squared Computing, Salem, OR, USA).

### 2.6. Definition and Calculation of the Impulse

**Figure 1:**
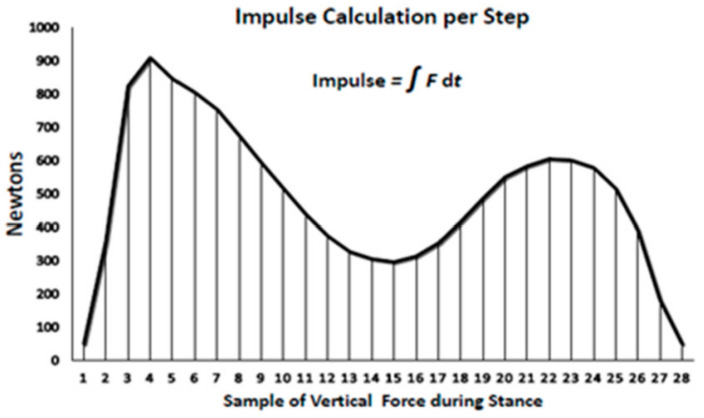
Calculation of impulse or force–time integral per step.

Impulse, or the force–time integral, calculated for the number of effective steps at a given speed [22], is the area under the force–time curve for each step, which depicts the sum of the effect of force over time (Figure 1) during the loading portion of the gait cycle. 

Impulse, measured in newtons per second (N·s) is determined [22] using the trapezoid rule over the duration of the stance phase of walking [52] when the total ground reaction force is greater than 50 N from the start to the end of each step. The individual samples of force are calculated during the stance and are multiplied by 0.02 s and then summed to calculate the impulse per step. The impulse measure based on other force-time measures, is applied in clinical studies where the health outcome is changed by a modification of either the force or the duration of its application [53]. In the context of our study, the impulse represents the duration of the effective exercisestimulus at a given walking speed for the suppression of bone mineral loss [20] or for the stimulation of fat loss.

### 2.7. Statistical Analysis

Data are presented as means and SEMs. Using the mixed-model repeated-measures analysis of variance by the Statistical Analysis System (SAS version 9.4, SAS Institute, Cary, NC, USA), the treatment, time, and their interactions were analyzed as between-subject effects, and the values for each of the 25 subjects and the study duration within the speed subgroups as a within-subject random intercept. Between-group and speed subgroup differences after 15 and 30 weeks of exercise were calculated with the *t*-test procedure using Tukey–Kramer adjustment for multiple comparisons. Dependent variables were the total body and limb fat and LBM by DXA scans, percent body fat estimates by anthropometric equations of Teran [49] and Jackson [50], and anthropometric measurements of regional fat in the form of SC fat SKFs and Circs of select peripheral body sites. 

## 3. Results

### 3.1. Exercise Outcome

Walking speed was 6.6 km/h at about 119% of VT in fast walkers and 5.5 km/h at 101.6% of VT for slow walkers. Walking speed between the two groups expressed in km/h and as a percent of VT was significantly higher (18.5 and 18.7%, respectively) in the fast compared to slow walking groups. In addition, the relative effort at VT was 31% higher in fast compared to slow walkers. Throughout the entire 30 weeks, the slow walkers maintained an average relative intensity of between 101.3% and 101.8% of VT at speeds of between 5.3 and 5.4 km/h. During the first 15 weeks of training, high-speed walkers maintained an average speed of 122.5% of VT at 6.6 km/h, but those subjects who continued through week 30 of the study reduced their speeds to 115.5% of VT during the second 15 weeks (F = 5.30, t = 2.94, df 16.1, *p* = 0.0422). Because of this reduction in walking speed, the relative effort of fast walkers at VT declined to 20.49 ± 3.04 mL O_2_/kg·min vs. 15.75 ± 1.86 in slow walkers, a decline of about a 2 mL O_2_/kg·min, which made the group difference non-significant. Since the 4.8 km distance travelled by both groups was the same, the duration of daily exercise session was 21.6% longer in the slow compared to fast walkers, but EEE was isocaloric and equal independently of speed as expected from the data of others [44] (Table 3). 

There was no difference in compliance with the study protocol as a percent of assigned distance between the 15-week and 30-week groups overall (F = 0.03, df = 23.7, *p* = 0.86), over the duration of training (F = 1.66, df = 18.5, *p* = 0.21) or in terms of minutes walked per week (F = 2.48, df = 23.5, *p* = 0.13; F = 1.09, df = 17.3, *p* = 0.31), but the mean number of days walked per week was four rather than the assigned five.

### 3.2. Effects of 15 and 30 Weeks of Walking, Independent of Speed, on Whole-Body Fat and Lean Body Mass

#### 3.2.1. Effects of 15 and 30 Weeks of Walking, Independent of Speed, on Body Fat and Lean Body Mass Measured by DXA and Anthropometric Measures 

Table 4 presents the overall effect of walking, independently of speed, on grams of body fat measured by DXA scans in the 15-week and 30-week groups. No fat loss occurred after 15 weeks of walking in the 15-week group, but there was a significant time effect for both the fat loss and percent fat loss. The 30-week group lost 0.5 kg (1.4%) of fat after 15 weeks of walking and 2 kg (5.7%) after 30 weeks, with only a significant effect of time (F_(df=1/14)_ = 3.52, *p* = 0.044) and no effect of treatment (F_(df=1/14)_ = 3.31, *p* = 0.09) or of an interaction between treatment and time (F = 1.25, *p* = 0.30). However, while there was only a 1.8% percent fat loss after 15 weeks in the 30-week group, a 3.9% percent fat loss after 30 weeks of walking was significant (F_(df=1/14)_ = 6.05, *p* = 0.028) along with a significant effect of time (F = 4.59, *p* = 0.02). Estimations of percent body fat change by the anthropometric Jackson 7 equation again revealed no significant fat loss after 15 weeks in the 15-week group. There was about half as much fat loss in the 15-week group compared to the 30-week groups with the other two equations (0.9% vs. 1.6% and 1% at 15 and 30 weeks, respectively, with Jackson 11, and 1.1% vs. 2.3 and 2.1% with the Teran equation). There also were significant changes in limb fat and lean body mass (LBM) in both 15-week and 30-week groups (Table 4). In the 15-week group, arm fat decreased by 5.8% and also decreased by 3% and 5.9% after 15 and 30 weeks of walking, respectively. Leg fat and percent leg fat only decreased in the 30-week group, 0.4 and 3.5% by week 15, and 1.1% and 1.9% by week 30, respectively. There was only a significant effect of time for the total and percent total decrease in LBM in the 15-week group. In the 30-week group, total and percent total LBM significantly increased by 1.1 and 0.5% after 15 and 30 weeks of walking. Corresponding increases in percent total LBM in this group were 1.6 and 3.3%, respectively. Percent arm LBM in this group increased 1.7 and 4.4%, and percent leg LBM increased 1.1 and 1.9%, respectively. Both percent leg LBM and percent arm LBM also significantly increased in the 30-week groups.

#### 3.2.2. Effects of 15 and 30 Weeks of Walking, Independent of Speed, on Skinfold and Circumference Measurements 

Table 5 presents the overall effect of walking, independently of speed, on SKFs and Circs of select body parts. Three 15-week subjects and one 30-week subject received no anthropometric measurements. None of the SKFmeasurements were significant after 15 weeks of walking in the 15-week group. By contrast, in the 30-week group, all SKF measurements significantly changed, although not in a consistent fashion, after both 15 and 30 weeks of walking. SKF at the abdomen at the umbilicus increased by 1.4% at week 30, suprailiac SKF decreased after 15 weeks of walking but regained its original thickness by week 30, and thigh SKF decreased by 3.5% after 15 weeks and a total of 3.8% by week 30. Circ measurements declined significantly in both the 15-week and 30-week groups except for the calf Circ, which increased in both groups. In the 15-week group, abdominal Circ declined by 1.8%, upper arm by 0.4%, gluteal site by 0.6%, thigh by 0.2%, and the sum of seven Circs by 0.6%. In the 30-week group, the declines in Circs after 15 and 30 weeks of walking were, respectively, 2.9 and 3.3% for the abdomen, 0.2 and 1.3% for the upper arm, 0.8 and 0.5% for the gluteal site, 0.4 and 1.2% for the thigh, and 1.1 and 1.4% for the sum of seven Circs. 

### 3.3. Effects of Walking Speed on Total Fat and Lean Body Mass after 15 and 30 Weeks of Walking Measured by DXA and Anthropometric Equations

**Figure 2:**
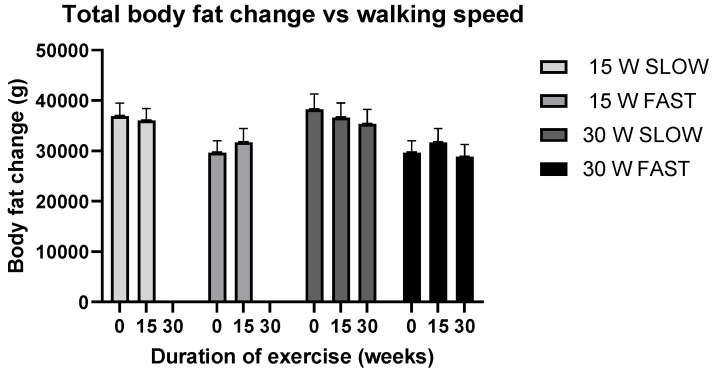
Effects of walking speed, measured by DXA on total body fat after 15 and 30 weeks of walking.

**Figure 3:**
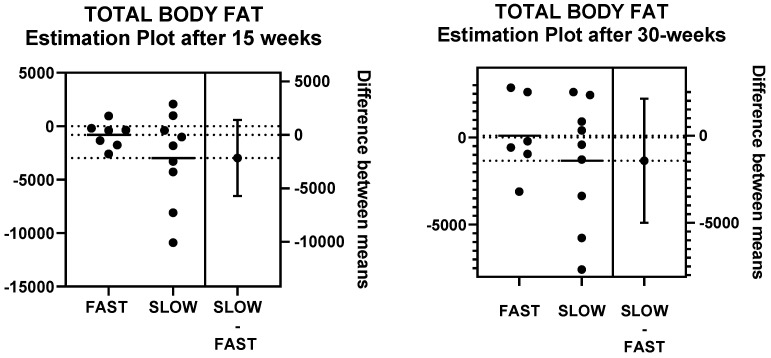
Estimation plots of total body fat as a function of walking speed after 15 and 30 weeks.

The effects of walking speed on total fat and lean body mass after 15 and 30 weeks of walking, measured by DXA, are shown in Table 6 and Figure 2 and Figure 3. By DXA measurements, neither fat nor its percentage changed as a function of speed in the 15-week group. In the 30-week group, fat loss of 4.2% and 7.5% after 15 and 30 weeks of walking, respectively, approached significance, and −3% and −4.2% changes in percent body fat after 15 and 30 weeks of walking were significant (Table 6). The difference in the effect of walking speed in both 15-week and 30-week groups is noticeable in Figure 2, showing that body fat in fast walkers either increased after 15-weeks of walking or increased at 15 weeks and then declined after 30 weeks of walking. By contrast, the 30-week slow group showed progressive body fat decreases during the 30 weeks of walking. Figure 3 is an estimation plot showing that the magnitude of absolute weight-loss values in individual slow walkers is greater than in fast walkers, although the range and number of subjects on either side of the mean value are equal. Estimates of body fat by Jackson and Teran equations, which contain SKF and Circ measurements, showed opposite fat changes as a function of walking speed compared to DXA measurements. At slow walking speed, both of the Jackson equations and the Teran equation estimated significant overall increases in body fat. In the 15-week group, there was an 0.6% increase in Jackson 7 equation, an 0.2% loss in Jackson 11, but a 0.2% fat gain in Teran. In the 30-week group at 15 and 30 weeks of walking, there was a 0.5 and 1.9% gain and a 0.7 loss followed by a 0.7% gain in Jackson equations 7 and 11, and a 0.1% loss followed by a 0.5% gain in Teran, respectively. By contrast, at fast walking speed, estimates of body fat by Jackson and Teran equations showed overall losses in both 15-week and 30-week groups. In the 15-week group, fat losses at a fast speed were 1.3 and 1.9% with Jackson 7 and 11 equations, respectively, and 2.8% with the Teran equation. In the 30-w group after 15 and 30 weeks of fast walking, body fat changes were estimated to produce 0.5 and 1.9% losses and a 0.7% loss and 0.7% gain, respectively, with Jackson 7 and 11 equations and a 0.1% loss and 0.5% gain with the Teran equation. 

**Figure 4:**
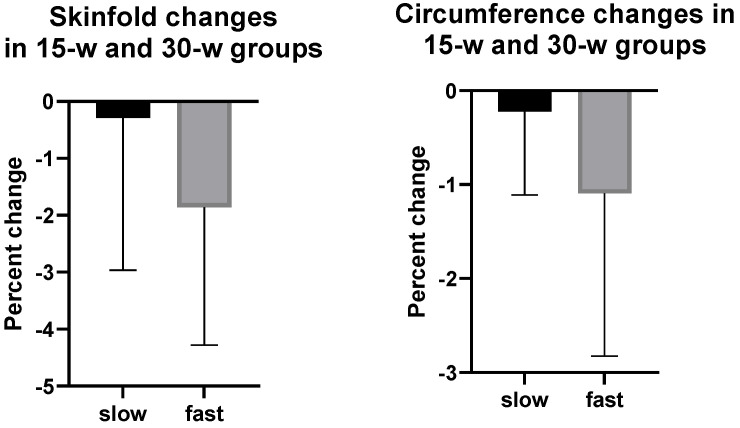
Skinfold and circumference percent changes in 15-week and 30-week groups.

The effects of walking speed on anthropometric SKF and Circ measurements of regional fat and lean body mass after 15 and 30 weeks of walking are shown in Table 6 and Figure 4. Individual SKF measurements revealed a similar pattern of fat gain for the abdomen and the sum of SKFs in slow walkers in both the 15-week and 30-week groups (15 w SKFs 1.7% and 1.5% gains, respectively, and 30 w 1.4% and 4.9% and 1.8% and 2.6% gains, respectively). By contrast, fast walkers showed opposite fat changes for abdominal SKFs and the sum of five SKF changes. Both the abdominal SKFs and the sum of SKFs decreased by 2.7% and 1.5%, respectively, in 15-week fast walkers. In 30-week fast walkers, the 30-week fat loss was estimated to be 1.4% for the abdomen and 3.9 and 2.4% for the sum of SKFs after 15 and 30 weeks of walking. Abdominal, gluteal, and thigh Circs showed no differential effect of speed, and all displayed losses after both 15 weeks and 30 weeks of walking. They were abdominal, gluteal, and thigh Circs, all showing between 0.3 and 3% declines.

Upper arm Circs reflected changes in SC fat, while changes in the calf Circ likely reflected changes in LBM as the calves were engaged in walking. In the 15-week group, upper arm Circ did not change in slow walkers and decreased by 0.9% in the fast walkers. In the 30-week group after 15 and 30 weeks of walking, upper arm Circ decreased by 1% and 0.9% by week 30. Calf Circ, on the other hand, increased in the 15-week group at both the slow (1.4%) and fast (2.5%) speeds. Corresponding increases in calf Circ in the 30-week group were 3.2%% and 0.2% after 15 and 30 weeks of slow walking, and 1.9 and 2.7% after corresponding durations of fast walking. LBM did not change in the 15-week group in total body, percent body, and percent arm and leg values, regardless of the speed. However, all three percent LBM measurements in the 30-week group changed as a function of walking speed. After 15 weeks of walking, slow walkers in the 30-week group gained about twice as much percent total-body LBM compared to fast walkers, 2.9 vs. 1.2%. After 30 weeks of walking, slow walkers in the 30-week group again increased their LBM to a greater extent than fast walkers (4.2 vs. 2.7%). This pattern continued for the change in percent arm LBM (4.8% gain for slow walkers vs. 0.2% loss for fast walkers after 15 weeks, and 5.6% gain for slow walkers vs. 3.4% gain for fast walkers after 30 weeks. Percent leg LBM increases were smaller and similar in magnitude after 15 weeks (1.8% at both speeds) and after 30 weeks (2.1 and 1.7% for slow and fast walkers, respectively).

### 3.4. Dietary Intake

Three-day food diaries were available for 11 slow and 6 fast walkers in the 15-week group and for 9 and 7 subjects, respectively, in the 30-week group. Although it appeared that food intakes in slow walkers declined, and in fast walkers increased after 15 and 30 weeks of walking relative to their baseline values, differences in the reported food intakes between fast and slow walkers at either of the three time periods were not significantly different (Table 7).

## 4. Discussion

This study had two aims. The first one was to examine the efficacy of exercise in producing total and abdominal fat loss in postmenopausal women and provide information that could alleviate some of the health risks associated with overweight and obesity in general [2,3,4,5,6,7,8,9] and with obesity in postmenopausal women in particular [16,17,18,19], given the weight gain and redistribution of body fat from peripheral to more deleterious central sites associated with menopause [13,14,15,16,17,18,19,54]. We focused on the speed of movement as the likely variable to affect both the rate of total and SC and VF fat loss and the retention of LBM, and also because it was shown to be required for the suppression of bone mineral loss in the same subjects being analyzed in the present study [20]. The second aim was to capitalize on the special opportunity to compare the effects of the speed of exercise and duration of exercise bouts at a given speed on fat loss on one hand, and bone mineral loss with the same subjects in the same study on the other hand. Here, the goal was to determine whether fat loss and the suppression of bone mineral loss required the same exercise features, as we were aware that some physiological systems such as changes in insulin sensitivity respond to unexpected aspects of exercise [23]. In a series of three studies [20,21,22], we showed that mineral loss is suppressed in postmenopausal women by higher speeds of walking (above 6 km/h) and by 40- to 45-min-long impulses (exercise bouts at a given speed), but not by the shorter 20-min exercise impulses or the lower walking speed of about 5.5 km/h. Reviewing studies that indicated that a lower walking speed and exercise intensity produce greater total body fat loss than higher exercise intensity [32,35,36,43], we first hypothesized that women walking at about 100% of VT will lose more fat than women walking at about 120% of VT. In our second hypothesis, we were influenced by studies showing greater SC fat loss at a higher, rather than at a lower, intensity or speed of exercise [33,38,39,41,42] and posited that women walking at 120% of VT will lose more SC fat than women walking at 100% of VT. In the present study, exercise at both speeds was isocaloric because the speed of movement over the same 4.8 km distance expends the same energy [44].

### 4.1. Testing of Hypothesis 1: Lower-Speed and Longer Duration of Aerobic Exercise over a 4.8 km Distance Will Produce Greater Total Body Fat Loss Than Isocaloric Exercise at Higher-Speed

DXA data in Table 6 provide the answer to our question as to whether a difference in walking speed and duration of impulse affects total body fat loss in a similar way to how it affected bone mineral loss with the same postmenopausal subjects engaging in exercise with the same parameters. Exercise frequency was 4 days/w generating a weekly walking exposure of 19.2 km, the fast walking speed was 6.5 km/h and the slow speed was 5.5 km/h, the distance covered was 4.8 km at a constant EEE of about 300 kcal, and the training duration was 15 weeks for 25 subjects and 30 weeks for 16 subjects. In 25 subjects, 15 weeks of walking at a higher speed significantly suppressed the loss of bone mineral [20]. As Table 6 and Figure 2 show, after 15 weeks of walking, the same fast walkers did not lose any significant amount of total body fat. The group of subjects who carried out their walking for 30 weeks showed more distinct speed-associated changes in total body fat. The fast walkers in the 30-week group gained 1.24% after 15 weeks of walking but lost 2.75% by week 30. By contrast, slow walkers showed a progressive loss of total body fat over 30 weeks, with 4.2% by week 15 and 7.5% by week 30, which approached significance. Their percent fat change of 3% and 4.2% after 15 and 30 weeks of slow walking was significant. By these measures, exercise intensity and impulse specifications for total fat loss and the suppression of bone mineral loss were different, and the answer to the above question is the difference in walking speed and impulse duration affected total fat loss in an opposite way to how it affected the suppression of bone mineral loss in the same postmenopausal subjects [20].

These results raise the question of why the pattern of fat loss as a function of walking speed was different in the 15-week and 30-week groups (Table 6, Figure 2). Several variables could have contributed to divergent fat-loss results. The most likely are the role of starting body fat, size of necessary impulse or duration of the exercise bout, particular physiological requirements for bone mineral and body fat management, or simply a requisite number of study subjects to overcome the variance in the measurements of bone and body fat changes. Table 2 shows that the baseline body fat of slow walkers in the 15-week group was about 7.5 kg or 20% higher than in the fast walkers, a difference only approaching significance. However, the 5.9% difference between slow and fast walkers or the 12% increase in percent body fat reached significance. The contrast in the pattern of fat loss at two speeds may have been influenced by the difference in the baseline body fat between fast and slow subjects. A rare study [55], which compared fat loss in obese (38% body fat) and normal-weight (24% body fat) middle-aged women after 12 weeks of 4 days/w exercise at 75% of VO_2_ max supports this interpretation. Body fat decreased to a greater extent in the obese (*p* < 0.001) than in the normal-weight women (*p* < 0.05), though the 10 SKFs decreased in both groups with no between-group differences. A meta-analysis of studies on the effects of exercise on fat loss [56] confirmed that overweight and obese subjects lose more fat than lean subjects exercising at the same relative effort. The difference is attributed to greater compensatory mechanisms in lean compared to obese subjects, potentially consisting of changes in general physical activity or food intake in subjects of different body-fat categories. Considering the different levels of baseline fatness in fast and slow walkers in the present study, we view hypothesis 1 as supported. Slow walking after 30 weeks produced progressive and overall greater and earlier fat loss than fast walking.

Besides the possible influence of the baseline fat level, it is instructive to consider the possible role of the duration of daily activity at a given speed (impulse) or weekly exercise exposure when comparing the fat-loss results in the same subjects to the bone-mineral results. As already mentioned, the effective walking duration for the suppression of bone mineral loss was 40 to 45 min per session coupled with a walking speed above 6 km/h [20]. A longer 59-min impulse in women walking at about 5.5 km/h did not produce suppression of bone mineral loss. The explanation is that bone mineral change required a higher exercise intensity combined with a minimum of a 40- to 45-min exercise bout duration, as shortening the impulse time to 20 min was ineffective [22]. In the same women in this study, a slow walking speed of 5.4 km/h and a 54-min impulse was more effective at total-body fat loss than walking at 6.6 km/h for 44.7-min (Table 3). Different speed and impulse requirements with the same subjects and exercise paradigm indicate that bone and adiposity respond differently to the speed and duration of an exercise session. Bone required a minimum of 40–45 min of mechanical stimulation at a high walking speed to suppress its mineral loss, while fat loss appears to be responsive to oxidative utilization of predominantly lipid fuel at a slow walking speed in combination with a longer walking bout duration. 

While the combination of exercise intensity and speed affects fat loss and bone mineral metabolism in opposite ways, a greater duration of daily exercise impulses or duration of weekly exercise exposure facilitates fat loss at either a fast or slow speed. To suppress bone mineral loss, the daily impulse requires to be 40- to 45-min long in combination with fast walking speed. Effective fat loss at either slow or fast walking speed benefits from the prolongation of daily exercise impulse or weekly exposure to exercise. The benefit of a longer impulse in moderate-intensity exercise in facilitating total body fat loss was highlighted in a study [40] in which 168 overweight postmenopausal women walked briskly for a year, at a walking speed comparable to slower walking in the present study. After stratifying walkers by fitness and the duration of exercise bouts, total fat loss was highest (4.2%) for women who walked for more than 195 min/week, intermediate for exercise exposure of 136–195 min/week (2.4%), and lowest for those who walked less than 135 min/week (0.6%). The second study [41] demonstrated that prolonging the weekly exposure to high-intensity exercise also significantly increases total fat loss. In this study, overweight men and women who walked or jogged 32 km/week at 78% of VO_2_ max lost 4.8 kg of total fat mass compared to 2 kg total fat loss at the same relative intensity in subjects whose weekly exercise exposure was 19.2 km/week, the same as the weekly distance in our study. An impressive demonstration of the effectiveness of longer bouts at a high exercise intensity was shown in a study [57] in which young sedentary obese men expended 1100 kcal/day during 90-min exercise bouts. The exercise lasted 16 weeks and exposure was 5 days/w. Fat loss was 5.9 kg and LBM gain was 0.2 kg for a net weight loss of 5.7 kg. These studies also help explain why fast walkers in the present study started losing total body fat between weeks 15 and 30, after first gaining weight by week 15 (Table 6 and Figure 1). As high-intensity exercise training increases relative fitness, it also increases the capacity of aerobically trained individuals to utilize lipids as the metabolic fuel for exercise [58,59] at the expense of carbohydrates [60]. Comparisons of the magnitude of fat loss with other studies that provide longer impulses than the one implemented in our study suggest that the fat loss would have been greater had we provided in our study an impulse longer than the 54 min in slow walkers or the 44.7 min in the fast walkers, as they were limited by the assigned 4.8 km daily walking distance and 19.2-km weekly exercise exposure. 

The final general issue necessary to consider in the context of the effect of exercise on fat loss and exercise speed or intensity on fat loss in particular is that some expended energy is repartitioned toward synthesis of LBM. This process was described in a study [61] that quantified changes in fat mass and nitrogen sparing associated with sparing the material necessary for building the LBM during a 7-day caloric deficit of 1000 kcal/day. The energy drain was generated either by running at 80% VO_2_ max to expend 15 kcal/kg·day for a total of 1000 kcal, or dietary restriction producing an equivalent energy loss. Weight loss during the exercise week was 0.76 kg, significantly less than 2.16 kg produced by the dietary restriction week. The difference in fat loss during the 7-day caloric restriction was counterbalanced by significantly lower nitrogen loss during the exercise week (only 11 g) than the nitrogen loss (24.5 g) by dieting. This shows that EEE is partitioned between nitrogen sparing, a required element to build LBM at high exercise intensities on one hand, and loss of body fat on the other. Exercise is usually reported to produce significantly greater increases in LBM at high compared to low exercise intensities [30,31], but LBM increases are also reported to occur at both high and low intensities [28], and with a longer exercise impulse at the same intensities [32,62]. In the present study, changes in LBM as a function of overall exercise are reported in Table 4, and as a function of walking speed, in Table 6. Considering the 15-week and 30-week group overall responses, exercise produced increases in both the total and percent LBM in both groups (0.7% and 0.3%, respectively in the former group, and 0.5 and 3.3% at 30 weeks in the latter). Percent arm LBM increased by 1.5% in the 15-week group, and 2.2 and 4.4% at 15 and 30 weeks, respectively, in the 30-week group. Corresponding increases in percent leg LBM were less robust than in percent arm LBM (0.6% in the 15-week group and 1.1 and 1.9% in the 30-week group). Considering increases in LBM as a function of walking speed, no increases were seen in the 15-w group regardless of walking speed. In the 30-week group, increases in percent total, arm, and leg LBM were consistently and significantly higher is slow-walking than in fast-walking women.

The outcome of testing Hypothesis 1 suggests that overweight and obese individuals will benefit more from lower- than from high-intensity exercise if it is combined with longer impulses involving greater energy expenditure than provided in the 4.8 km distance in the present study. High-intensity exercise combined with long daily or weekly exercise exposures will produce increased fat losses, regardless of initial body fat level, if the training lasts long enough to increase relative fitness and associated increases in lipid fuel utilization. Either walking speed will also produce increases in LBM and counteract some of the declines that occur during the menopausal transition [54].

### 4.2. Testing of Hypothesis 2: Higher Exercise Speed Will Produce Greater Abdominal SC Fat Loss than Isocaloric Exercise at Lower Speed 

The importance of reducing abdominal subcutaneous and visceral fat in postmenopausal women has already been mentioned. During the postmenopausal transition (PT), which lasts about 7 to 10 years [54], central adiposity increases [12,13,14,15]. Android fat increases by 1.21% and visceral fat by 6.4% per year. Waist girth or Circ, a valid indirect measure of visceral fat [18,19], increases 0.55% per year during premenopause and postmenopause, and 0.96% per year during the PT [54]. Abdominal, and in particular visceral, adiposity is strongly linked to insulin resistance, type 2 diabetes, hypertension, dyslipidemia, sleep apnea, and other complications of obesity [13,14,15,16,17,18,19,63]. The distribution of body fat appears to produce greater health risks than the total amount of body fat [63]. For those reasons, abdominal SC fat and VF have been measured in a number of studies using anthropometric approaches as in our study, or more recently, with technologically more sophisticated imaging techniques. Circ measurements are considered valid estimates of visceral fat [18,19]. However, the inferences from anthropometrical approaches should be considered as only estimates of obesity, because there are instances where they produce fat losses that are greater than the more valid measurements of EEE [34,64]. We have formulated Hypothesis 2 on the basis of evidence that higher exercise intensity or walking speeds produces greater SC fat and VF losses than lower exercise intensity or slower walking speeds [33,38,39,41,42]. The results of our anthropometric measurements after overall exercise, independent of speed, are presented in Table 5. Discussion of abdominal SC fat and Circ was separated from measurements of other parts of the body because of the serious health consequences of central obesity [16,17,18,19,61]. As presented in Table 5, abdominal Circ measurements, without consideration of walking speed, showed significant losses in both the 15-week (1.8%) and 30-week groups (2.9%) after 15 weeks, and 3.3% after 30 weeks of walking. SKF measurements did not change in the 15-week group but increased significantly by 1.3% in the 30-week group after 30 weeks of walking. 

Table 6 presents changes in anthropometric measures as a function of walking speed. It shows that speed did not have a differential effect on abdominal Circ changes in the 30-week group, as their Circs declined by about 3% after 3 weeks of walking in both fast and slow walkers. These results therefore agree with the results and conclusions in a previous study [39] that shows declines in the abdominal VF in response to EEE, and not to variations in walking speed. However, the walking speed did differentially affect Circs in the 15-week group in that they declined by 3% at fast walking speed, but only 0.8% by slow speed. Further research with larger number of subjects will need to determine whether in leaner subjects of the 15-week group, Circ declines would equalize at either walking speed by 30 weeks of exercise., As was the case with the effects of overall exercise, independently of walking speed, abdominal SKFs were unaffected in the 15-week group at either speed. In the 30-week group, slow walkers gained 1.4% and 4.9% of abdominal SC fat after 15 and 30 weeks of walking, while fast walkers gained 6.5% abdominal SC fat after 15 weeks but lost 2.8% of it after 30 weeks of walking. The pattern of abdominal SC fat change in the 30-week fast walkers appears to mirror the total fat changes as a function of walking speed in this group (Table 6 and Figure 2). While we do not understand why the estimates of abdominal SC and VF appear to differ, it would appear that choosing a fast walking speed over a slow one would be more beneficial in reducing the health risks associated with central obesity. A fast walking speed reduces waist Circ, the surrogate estimate of VF, as effectively as a slow walking speed, and it also appears to reverse an initial SC abdominal fat gain at a fast walking speed with the fat loss after 30 weeks.

The effect of walking speed on the percent changes of SKFs at supra-iliac and thigh sites and of Circs at the upper arm, gluteal, and thigh sites are presented in Figure 4 and Table 6 for clarity. Figure 4 presents percent changes as a function of walking speed in individual SKFs and Circs and their sums in our study and Jackson and Teran equations in the 15-week and 30-week groups. The figure illustrates that fast walkers reduced their individual and summed SKFs and Circs about five-fold more than slow walkers despite the small number of end-point comparisons, large variance, and lack of significant differences. Supra-iliac and thigh measurements contributed to increased SKF variance, and the three equations to Circ variance. In contrast to our results with SC abdominal and VF, there were conspicuously higher Circ losses at these peripheral body sites after fast than slow walking.

Discussion of calf anthropometric measurements was also separated from measurements of other parts of the body because of the probability that they represent greater involvement of increases in LBM than of the loss of fat (Table 6). Both fast and slow walking in the 30-week group (but not in the 15-week group) were associated with a 1.8% increase in total leg mass measured by DXA. The additional 15 weeks of walking did not increase overall LBM much further than 2%. Calf circumferences initially increased more in slow than in fast walkers of the 30-week group (2.8 vs. 1.9%, respectively), but the gain was reversed during the next 15 weeks, when calf circumference declined to 0.2% in slow walkers, while fast walkers increased it to 2.7%. We conclude that fast walking produces greater gains in calf LBM in agreement with the preponderance of data on the stimulatory effects of the intensity of exercise on LBM gain, exemplified in two previous studies [30,31].

From the above data evaluations, Hypothesis 2 was not supported in its original form in that we did not find greater abdominal and visceral fat loss in fast compared to slow walkers. Instead, we confirmed the demonstration of others [39] that visceral fat is responsive to energy expenditure in general, rather than on the speed of walking or differences in exercise intensity.

Fast walking appeared to reduce individual SKFs and Circs and the sums of body parts other than abdomen and calf by 5-fold more than slow walking (Figure 4), but these changes did not reach significance. Calf mass measured by DXA increased to a similar extent at both exercise speeds, but calf circumference increased more consistently in fast walkers.

## 5. Study Limitations

The limitation of the study includes an issue of study design and the issue of requisite numbers of subjects to produce power for consistently significant outcomes. The design limitation was that we provided subjects with a relatively short walking distance of 4.8 km per daily session and only a 19.2 km weekly exercise exposure. This translated into a 54.5 min impulse for slow walkers and a 44.7-min impulse for fast walkers. Having more than one distance would have extended the range of impulses and strengthened our inferences regarding the efficacy of impulse length to increase fat loss in both slow and fast walkers. A change in starting body fat, which was a consequence of subject attrition after the first 15 weeks of walking, confounded our comparison of the effects of fast and slow walkers On total and regional fat loss The benefit of this limitation and error was that we were prompted to consider the level of baseline fatness as a likely cause for greater effectiveness of fat loss at a slower walking speed. If we had used DXA-determined body fat to match fast and slow walking groups, we would have had a stronger basis for inferences on the effects of speed, independently of starting body fat, on total fat loss. Measurements of total and regional body fat in the same subjects who provided strong and unambiguous evidence that higher walking speed suppresses bone mineral loss in postmenopausal women [20] made it clear that the variance in fat measurements over 15 weeks of walking is substantially higher than the variance in bone mineral measurements. It was clear from the data in Table 6 that the results would have been more clear-cut, and significant differences greater, if the number of study subjects was larger. At least one study has produced significant results on the effectiveness of higher speed on abdominal fat loss with subject numbers as low as in our 30-week group [33].

Despite not having subjects matched for baseline body fat, and not having a sufficiently large subject sample to achieve statistical significance in all measurements, we accomplished all of the study aims: We confirmed that walking a 4.8-km distance for 15 weeks by postmenopausal women produces some total body fat and regional fat loss, that walking at either speed significantly reduces abdominal VF estimated from abdominal Circs and thereby reduces the risk of morbidities associated with postmenopausal accumulation of central fat, and that a difference in the duration of impulse for the same exercise in the same subjects accounted for different outcomes in fat loss and suppression of bone mineral loss. The required impulse the suppression of bone mineral loss after 15 weeks of walking was 40 to 45 min at a speed above 6 km/h, and the effect was abolished by shortening it [22]. For fat loss, a longer 54-min impulse at a lower speed was more effective than the shorter 44.7-min impulse at a higher speed in the overweight 30-week group. However, the higher duration of the exercise session could have been beneficial for fat loss in fast walkers as well, since they demonstrated fat loss after 30 weeks of walking, most likely due to increased aerobic fitness and improved ability to utilize lipids as a fuel at higher exercise intensities.

## 6. Conclusions

This study compared the efficacy of the same exercise in the same 25 postmenopausal subjects on total and regional fat loss to previously published results on the suppression of bone mineral loss. Exercise was carried out at two different speeds associated with different impulses while walking over a 4.8 km distance. A fast speed was 6.6 km/h at about 120% of the ventilatory threshold (VT), and a slow speed was 5.5 km/h at 101.6% of the VT. The duration of the exercise bout (impulse) was 44.7 min for fast walkers and 54 min for slow walkers. Exercise energy expenditure was isocaloric at about 300 kcal per session and 12,000 kcal per week. We measured body composition with both DXA and anthropometric methods. Based on our reading of exercise-and-fat-loss literature, we first hypothesized that more total fat will be lost with a slow walking speed and longer impulse than with a fast speed and shorter impulse. Second, we hypothesized that more abdominal SC and visceral fat will be lost at fast walking speed. The first hypothesis was supported in the group of 16 subjects with inadvertently 20% lower starting body fat than the original group of 25 subjects. They progressively lost total body fat over 30 weeks of slow walking. The second hypothesis was not supported as posited, but the results confirmed conclusions of others that visceral fat, as estimated by waist Circ, is affected by the magnitude of energy expenditure and not the walking speed or intensity [39]. A faster walking speed led to a greater loss of individual SKFs and Circs and their sums in trunk sites other than the abdomen and calf, than the slow walking speed, but these changes did not reach significance. Calf mass measured by DXA increased to a similar extent at both exercise speeds, but calf Circ increased more consistently in fast walkers.

Our overall conclusion is that total body fat is lost through walking at both slow and fast speeds tested in our study, but the change is more clear, rapid, and greater in overweight–obese slow walkers, and therefore slow walking would be preferable in subjects with greater starting body fatness. Thus, in contrast to the efficacy of fast exercise with a 40- to 45-min impulse in the suppression of bone mineral loss during 15 weeks of exercise in postmenopausal women [20], a longer 54-min impulse at a lower speed of 5.4 km/h in our study produced greater total fat loss than the faster 6.6 km/h speed and a lower impulse of 44.7 min walking the same 4.8-km distance. Comparisons with other exercise-and-fat-loss studies suggest that a longer impulse increases total body fat loss at both slow [31] and high exercise speeds [38,41,42], and that our study design providing only a 4.8-km walking distance may have limited the magnitude of fat loss. Therefore, high-intensity exercise will produce superior weight loss, especially with a longer impulse, but with some delay shown in fast walkers in the 30-week group, necessary to increase aerobic fitness and produce the associated increase in the utilization of body lipids at higher exercise intensities [58,59,60]. Increased exercise energy expenditure at either walking speed produces equivalent declines in visceral fat (estimated in this study from abdominal Circ), and with sufficiently long impulses, should benefit the health of postmenopausal women by reducing this source of disabilities associated with central obesity.

## Figures and Tables

**Table 1:** Subject characteristics at baseline for the 15-week and 30-week groups.

Variable	15-Week Subjects	30-Week Subjects	t_(df=39)_; *p*
Subjects	25	16	
Age (years)	58.00 ± 0.99	58.75 ± 1.20	0.50; 0.6212
Years post menopause	11.88 ± 2.09	11.96 ± 2.45	0.02; 0.9805
Weight (kg)	77.42 ± 2.25	76.48 ± 2.79	0.27; 0.7922
BMI (kg/m^2^)	29.41 ± 0.74	29.36 ± 0.89	0.04; 0.9701
Total Fat (kg)	34.37 ± 1.82	34.46.± 2.26	0.032; 0.975
Total Fat (%)	45.90 ± 1.30	46.66 ± 1.67	0.361; 0.72
VO_2_ max (mL O_2_/kg/min)	29.77 ± 1.87	29.55 ± 2.35	0.07; 0.9426
VT (mL O_2_/kg/min)	16.54 ± 0.78	16.80 ± 0.98	0.21; 0.8319
Relative effort at VT (%VO_2_ max)	61.2 ± 5.4	60.9 ± 7.85	0.0327; 0.9742

**Table 2:** Subject characteristics at baseline for the fast and slow subjects in the 15-week and 30-week groups.

Variable	15-Week Slow Walkers	15-Week Fast Walkers	t_(df-23)_; *p*	30-Week Slow Walkers	30-Week Fast Walkers	t_(df=14)_; *p*
Subjects	*N* = 15	*N* = 10		*N* = 9	*N* = 7	
Age (years)	56.93 ± 1.10	59.60 ± 1.56	1.44; 0.1642	57.56 ± 1.57	60.29 ± 1.81	1.14; 0.273
Years post menopause	9.20 ± 2.03	15.71 ± 3.91	1.61; 0.1287	9.36 ± 2.25	15.0 ± 4.55	1.17; 0.267
Weight (kg)	79.33 ± 2.83	75.55 ± 3.66	1.04; 0.3071	78.33 ± 3.58	74.09 ± 4.25	0.77; 0.455
BMI (kg/m^2^)	30.12 ± 1.09	28.34 ± 0.76	1.10; 0.2440	30.37 ± 1.31	28.07 ± 1.05	1.32; 0.210
Total Fat (kg)	36.91 ± 2.56	30.57 ± 1.99	1.79; 0.0872	38.21 ± 3.10	29.64 ± 2.40	2.08; 0.056
Total Fat (%)	47.58 ± 1.87	43.37 ± 1.36	1.71; 0.1135	49.96 ± 2.05	42.41 ± 1.82	2.66; 0.020
VO_2_ max (mL O_2_/kg/min)	28.38 ± 2.75	31.85 ± 2.21	0.91; 0.3750	27.99 ± 3.17	32.01 ± 4.61	0.87; 0.398
VT (mL O_2_/kg·min)	16.70 ± 0.93	16.31 ± 1.42	0.24; 0.8123	16.84 ± 1.04	16.75 ± 2.06	0.04; 0.967
Relative effort at VT (mL O2/kg·min)	57.19 ± 5.53	65.10 ± 5.30	1.21; 0.2386	57.71 ± 7.43	62.28 ±7.39	0.55; 0.590

**Table 3:** Exercise outcomes for fast and slow subjects in 15-week and 30-week groups.

Variable	15-Week Slow Walkers	15-Week Fast Walkers	t_(df=23)_; *p*	30-Week Slow Walkers	30-Week Fast Walkers	t_(df=14)_; *p*
Subjects	*N* = 15	*N* = 10		*N* = 9	*N* = 7	
Walking speed (km/h)	5.45 ± 0.11	6.60 ± 0.36	3.584; 0.0016	5.35 ± 0.14 **	6.5 ± 0.16 *	5.32; 0.0001
Walking speed (% VT)	101.28 ± 1.64	122.46 ± 1.58	8.98; <0.0001	101.8± 1.95 **	118.6 ± 3.2 *	4.65; 0.0004
Relative effort at VT (mL O_2_/Kg·min)	17.53 ± 1.16	23.76 ± 1.69	3.14; 0.0047	17.19 ± 1.58 * 15.75 ± 1.86 #	24.32 ± 2.37 * 20.49 ± 3.04 #	2.59; 0.0213
Session duration (min)	53.51 ± 1.1	44.23 ± 1.37	5.32; <0.0001	54.44 ± 0.72 **	45.16 ± 2.16 **	4.51; 0.0005
EEE/session (kcal)	303.12 ± 10.7	285.44 ± 10.78	1.54; 0.2748	297.87 ± 12.08 **	279.15 ± 8.3 *	1.20; 0.249

* Values obtained after first 15 weeks of walking, ** Values are means of 15 and 30 weeks of walking, # Values obtained for the second 15 weeks of walking.

**Table 4:** Effects of 15 and 30 weeks of walking, independent of speed, measured by DXA and anthropometric equations, on total and regional fat and lean body mass.

DEXA Measurements	15-Week Group		30-Week Group			Treat F_(df=1/23)_; *p* Time F_(df=2/36.1)_; *p* Inter F_(df=2/36.1)_; *p*	Treat F_(df=1/14)_; *p* Time F_(df=2/27)_; *p* Inter F_(df=27)_; *p*
	Baseline	15 Weeks	Baseline	15 Weeks	30 Weeks	15-Week Group	30-Week Group
Subjects	*N* = 25	*N* = 25	*N* = 16	*N* = 16	*N* = 16	*N* = 25	*N* = 16
Body Fat (g)	34,372.12 ± 1816.08	34,387.21 ± 1832.88	34,463.81 ± 2257.66	33,967.53 ± 2309.54	32,488.31 ± 2069.84	Treat 2.15; 0.156 Time 3.40; 0.044 Inter 1.75; 0.1883	Treat 3.31; 0.09 Time 3.52; 0.044 Inter 1.25; 0.302
Body Fat (%)	45.90 ± 1.30	45.74 ± 1.32	46.66 ± 1.67	45.80 ± 1.79	44.82 ± 1.65	Treat 2.27; 0.145 Time 3.57; 0.038 Inter 0.55; 0.581	Treat 6.05; 0.028 Time 4.59; 0.02 Inter 0.4; 0.67
Arm fat (g)	3200.44 ± 356.30	3016.17 ± 247.29	2895.56 ± 273.89	2878.20 ± 237.79	2710.31 ± 229.58	Treat 1.80; 0.192 Time 1.04; 0.3623 Inter 3.31; 0.048	NS
Arm fat (%)	42.57 ± 1.64	41.72 ± 1.50	42.71 ± 1.61	41.45 ± 1.47	40.21 ± 1.68	NS	Treat 4.69; 0.048 Time 2.48; 0.102 Inter 0.82; 0.45
Leg fat (g)	12,937.00 ± 766.86	13,289.08 ± 792.55	13,164.88 ± 1047.86	13,218.07 ± 1068.89	12,708.81± 885.30	NS	Treat 4.86; 0.045 Time 1.62; 0.217 Inter 1.69; 0.20
Leg fat (%)	49.18 ± 1.40	49.50 ± 1.53	50.12 ± 1.98	49.57 ± 2.22	49.18 ± 1.99	NS	Treat 6.57; 0.023 Time 1.16; 0.327 Inter 0.13; 0.883
Total LBM (g)	39,369.36 ± 736.89	39,655.00 ± 794.18	38,396.06 ± 789.36	38,833.67 ± 949.42	38,590.56 ± 1127.76	Treat 2.15; 0.156 Time 3.40; 0.044 Inter 1.75; 0.1883	Treat 4.86; 0.045 Time 1.62; 0.217 Inter 1.69; 0.20
Total LBM (%)	54.10 ± 1.30	54.26 ± 1.32	53.34 ± 1.72	54.20 ± 1.79	55.1 ± 1.65	Treat 2.27; 0.145 Time 3.57; 0.038 Inter 0.55; 0.581	Treat 6.05; 0.028 Time 4.59; 0.019 Inter 0.4; 0.673
Arm LBM (%)	57.43 ± 1.64	58.28 ± 1.50	57.29 ± 1.61	58.55 ± 1.47	59.79 ± 1.68	NS	Treat 4.69; 0.048 Time 2.48; 0.102 Inter 0.82; 0.451
Leg LBM (%)	50.82 ± 1.40	50.50 ± 1.53	49.88 ± 1.98	50.43 ± 2.22	50.83 ± 1.99	NS	Treat 6.57; 0.023 Time 1.16; 0.328 Inter 0.13; 0.883
**Anthropometric Equations**	**15-Week Group**		**30-Week Group**			**Treat F_(df=1/20)_; *p*** **Time F_(df=2/32.1)_; *p*** **Inter F_(df=2/32.1)_; *p***	**Treat F_(df=1/13)_; *p*** **Time F_(df=2/25)_; *p*** **Inter F_(df=25)_; *p***
	**Baseline**	**15 Weeks**	**Baseline**	**15 Weeks**	**30 Weeks**	**15-Week Group**	**30-Week Group**
Subjects	*N* = 22	*N* = 22	*N* = 15	*N* = 15	*N* = 15	*N* = 22	*N* = 22
Jackson 7 body fat (%)	37.44 ± 1.14	37.32 ± 1.31	37.18 ± 1.34	36.83 ± 1.62	36.93 ± 1.61	NS	Treat 10.57, 0.006 Time 0.31, 0.733 Inter 0.38, 0.685
Jackson 11 body fat (%)	38.45 ± 1.04	38.09 ± 1.16	38.60 ± 1.33	38.00 ± 1.53	38.20 ± 1.44	Treat 5.40, 0.031 Time 0.56, 0.579 Inter 0.22, 0.800	Treat 12.2, 0.004 Time 0.72, 0.496 Inter 0.19, 0.829
Teran body fat (%)	38.22 ± 0.94	37.80 ± 1.16	38.58 ± 1.22	37.69 ± 1.62	37.78 ± 1.46	Treat 5.66, 0.027 Time 1.17, 0.324 Inter 0.60, 0.554	Treat 8.34, 0.013 Time 2.05, 0.15 Inter 1.21, 0.314

**Table 5:** Effects of 15 and 30 weeks of walking, independent of speed, on anthropometric measurements of abdominal and peripheral skinfolds and circumferences.

Anthropometric Measurements	15-Week Group		30-Week Group			Treat F_(df=1/20)_; *p* Time F_(df=2/32.1)_; Inter F_(df=2/32.1)_; *p*	Treat F_(df=1/13)_; *p* Time F_(df=2/25)_; Inter F_(df=25)_; *p*
	Baseline	15 Weeks	Baseline	15 Weeks	30 Weeks	15 Weeks	30 Weeks
Subjects	*N* = 22	*N* = 22	*N* = 15	*N* = 15	*N* = 15	*N* = 22	*N* = 15
**Skinfolds (mm)**							
Abdomen at umbilicus	44.29 ± 2.17	44.22 ± 2.06	44.16 ± 2.51	44.18 ± 2.46	44.75 ± 2.81	NS	Treat 8.21, 0.013 Time 0.09, 0.913 Inter 0.31, 0.733
Supra-iliac	24.96 ± 1.73	24.95 ± 1.81	24.23 ± 2.14	23.87 ± 2.07	24.20 ± 2.15	NS	Treat 5.36, 0.038 Time 0.08, 0.923 Inter 0.52, 0.601
Thigh	46.50 ± 2.08	46.02 ± 2.90	46.05 ± 2.67	44.50 ± 3.87	44.28 ± 3.28	NS	Treat 6.49, 0.0245 Time 1.26, 0.302 Inter 0.97, 0.393
Sum of 5 SKFs	165.46 ± 7.17	165.75 ± 8.21	164.29 ± 8.17	163.33 ± 9.76	163.73 ± 9.44	NS	Treat 7.53, 0.017 Time 4.67, 0.019 Inter 0.30, 0.747
**Circumferences (cm)**							
Abdomen at umbilicus	103.28 ± 2.59	101.39 ± 2.40	105.51 ± 3.21	102.42 ± 3.30	102.05 ± 3.38	Treat 4.95, 0.038 Time 2.06, 0.144 Inter 0.45, 0.64	Treat 7.53 0.017 Time 4.67, 0.019 Inter 0.30, 0.747
Upper arm	30.67 ± 0.64	30.56 ± 0.63	30.83 ± 0.76	30.78 ± 0.78	30.44 ± 0.80	Treat 4.75, 0.041 Time 1.88, 0.169 Inter 1.09, 0.035	NS
Calf	38.31 ± 0.64	39.03 ± 0.57	38.01 ± 0.77	38.71 ± 0.67	38.48 ± 0.73	Treat 1.10, 0.306 Time 7.54, 0.002 Inter 3.99, 0.023	Treat 0.95, 0.347 Time 4.26, 0.0026 Inter 3.05, 0.065
Gluteal	110.94 ± 1.69	110.32 ± 1.59	111.85 ± 2.26	110.94 ± 2.11	111.28 ± 1.89	Treat 6.59, 0.018 Time 0.38, 0.687 Inter 0.36, 0.701	Treat 7.65, 0.016 Time 0.47, 0.628 Inter 0.38, 0.689
Thigh	63.60 ± 1.07	63.45 ± 1.06	63.58 ± 1.30	63.36 ± 1.42	62.79 ± 1.41	Treat 5.49, 0.03 Time 1.66, 0.206 Inter 0.08, 0.92	Treat 5.60, 0.034 Time 1.59, 0.223 Inter 0.16, 0.852
Sum of 7 Circs	462.44 ± 6.56	459.75 ± 6.47	465.83 ± 8.9	460.85 ± 8.55	459.56 ± 8.18	Treat 5.58, 0.028 Time 1.99, 0.153 Inter 0.64, 0.536	Treat 6.79, 0.022 Time 3.08, 0.0638 Inter 0.72, 0.497
Waist-to-hip ratio at umbilicus	0.93 ± 0.02	0.918 ± 0.015	0.94 ± 0.019	0.922 ± 0.021	0.916 ± 0.024	NS	Treat 1.66, 0.22 Time 4.85, 0.017 Inter 0.21, 0.816

**Table 6:** Effects of walking speed on total and regional fat distribution after 15 and 30 weeks of walking measured by DXA and anthropometric methods.

		**15-Week Group**		**30-Week Group**			**15 Weeks**	**30 Weeks**
	**DXA Measurements**	**Baseline**	**15 Weeks**	**Baseline**	**15 Weeks**	**30 Weeks**	**Slow vs. Fast** **t(_df=28_); *p***	**Slow vs. Fast** **t(_df=16_); *p***
	**Subjects**	***N* = 15**	***N* = 15**	***N* = 9**	***N* = 9**	***N* = 9**		
**Slow walkers**	Body fat (g)	36,908.0 ± 2564.7	36,014.7 ± 2386.7	38,213.4 ± 3101.6	36,604.6 ± 2899.5	35,335.3 ± 2903.4	1.47; 0.1561	1.82; 0.0904
	Body fat (%)	47.6 ± 1.9	47.0 ± 1.7	50.0 ± 2.1	48.5 ± 1.9	47.9 ± 2.1	1.51; 0.1455	2.46; 0.0275
	Total LBM (g)	39,406.53 ± 1010.39	39,304.27 ± 1025.85	37,314.11 ± 923.53	37,338.33 ± 1183.04	37,525.56 ± 994.17	0.03; 0.9785	1.35; 0.1987
	Total LBM (%)	52.42 ± 1.87	53.01 ± 1.66	50.04 ± 2.05	51.48 ± 1.92	52.11 ± 2.14	1.51; 0.1455	2.46; 0.0275
	Arm LBM (%)	55.48 ± 2.57	57.51 ± 2.15	54.34 ± 2.18	56.93 ± 1.77	57.16 ± 2.26	1.25; 0.2243	2.17; 0.0479
	Leg LBM (%)	48.74 ± 1.83	48.52 ± 1.72	45.99 ± 2.10	46.81 ± 2.14	46.94 ± 2.13	1.89; 0.0711	2.56; 0.0225
	**Anthropometric equations (% fat)**							
	Subjects	*N* = 12	*N* = 12	*N* = 7	*N* = 7	*N* = 7		
	Jackson 7 body fat	39.09 ± 1.48	39.31 ±1.57	40.31 ± 1.22	40.50 ± 1.56	41.06 ± 0.84	1.75; 0.0961	3.25; 0.0064
	Jackson 11 body fat	40.39 ± 1.42	40.30 ± 1.43	41.83 ± 1.34	41.54 ± 1.50	42.11 ± 0.87	2.32; 0.0308	3.49; 0.0040
	Teran body fat	40.01 ± 1.33	40.09 ±1.43	41.25 ± 1.61	41.21 ± 1.79	41.44 ± 1.07	2.38; 0.0274	2.89; 0.0128
	**Skinfolds (mm)**							
	Subjects	*N* = 12	*N* = 12	*N* = 7	*N* = 7	*N* = 7		
	Abdomen 2	46.41 ± 2.35	47.20 ± 2.31	48.96 ± 1.20	49.63 ± 1.91	51.38 ± 1.54	1.56; 0.1352	2.87; 0.0133
	Supra-iliac	27.12 ± 1.97	26.71 ± 2.06	28.49 ± 1.75	25.96 ± 1.67	28.39 ± 2.05	1.30; 0.2071	2.31; 0.0376
	Thigh	50.09 ± 2.68	50.04 ± 3.27	51.79 ± 2.66	50.96 ± 3.80	51.61 ± 1.50	1.72; 0.1001	2.55; 0.0245
	Sum of 5 SKFs	173.37 ± 9.84	175.93 ± 10.79	180.69 ± 8.83	183.85 ± 11.3	185.42 ± 7.12	1.30; 0.2084	2.66; 0.0197
	**Circumferences (cm)**							
	Subjects	*N* = 12	*N* = 12	*N* = 7	*N* = 7	*N* = 7		
	Abdomen 2	107.41 ± 3.35	106.5 ± 2.84	112.38 ± 3.55	110.55 ± 3.91	109.21 ± 3.12	2.22; 0.0377	2.74; 0.0167
	Upper arm	31.86 ± 0.94	31.87 ± 0.85	32.21 ± 1.20	32.97 ± 01.19	31.89 ± 1.09	2.18; 0.0414	2.13; 0.0526
	Calf	39.11 ± 0.87	39.65 ± 0.75	38.86 ± 1.22	40.12 ± 1.12	38.93 ± 1.16	1.05; 0.3057	2.37; 0.0341
	Gluteal	114.27 ± 2.45	113.71 ± 2.12	116.38 ± 2.91	115.22 ± 2.73	115.76 ± 2.11	2.57; 0.0184	2.77; 0.0160
	Thigh	65.68 ± 1.17	65.49 ± 0.97	66.09 ± 1.50	66.18 ± 0.88	65.64 ± 1.40	2.34; 0.0295	0.98; 0.3470
	Sum of 7 Circs	475.4 ± 9.41	473.6 ± 8.0	483.8 ± 12.5	478.7 ± 10.6	477.8 ± 9.7	2.36; 0.0284	2.61; 0.0218
	W/H at umbilicus	0.94 ± 0.02	0.94 ± 0.02	0.97 ± 0.01	0.96 ± 0.02	0.94 ± 0.03	0.96; 0.3506	1.29; 0.2200
		**15-Week Group**		**30-Week Group**				
	**DXA Measurements**	**Baseline**	**15 Weeks**	**Baseline**	**15 Weeks**	**30 Weeks**		
	**Subjects**	***N* = 10**	***N* = 10**	***N* = 7**	***N* = 7**	***N* = 7**		
**Fast Walkers**	Body fat (g)	29,643.0 ± 2397.6	31,674.7 ± 2769.5	29,642.9 ± 2397.6	30,012.0 ± 3456.3	28,827.9 ± 2461.8		
	Body fat (%)	42.4 ± 1.8	43.7 ± 2.1	42.3 ± 2.8	41.7 ± 2.8	40.9 ± 1.8		
	Total LBM (g)	39,313.60 ± 1112.40	40,239.56 ± 1303.02	39,787.14 ± 1141.27	41,076.67 ± 1128.59	39,959.86 ± 2240.05		
	Total LBM (%)	56.63 ± 1.36	56.33 ± 2.12	57.59 ± 1.82	58.28 ± 2.83	59.13 ± 1.76		
	Arm LBM (%)	60.36 ± 0.98	59.57 ± 1.82	61.09 ± 1.24	60.97 ± 2.39	63.17 ± 2.00		
	Leg LBM (%)	53.93 ± 1.86	53.79 ± 2.69	54.89 ± 2.46	55.87 ± 3.72	55.81 ± 2.75		
	**Anthropometric equations (% fat)**							
	Subjects	*N* = 10	*N* = 10	*N* = 7	*N* = 7	*N* = 7		
	Jackson 7 body fat	35.46 ± 1.63	34.99 ± 2.01	33.59 ± 1.73	32.64 ± 2.08	32.80 ± 2.17		
	Jackson 11 body fat	36.12 ± 1.22	35.45 ± 1.58	34.91 ± 1.48	33.96 ± 1.92	34.29 ± 1.72		
	Teran body fat	36.07 ± 1.01	35.05 ± 1.54	35.53 ± 1.03	33.67 ± 1.96	34.13 ± 1.87		
	**Skinfolds (mm)**							
	Subjects	*N* = 10	*N* = 10	*N* = 7	*N* = 7	*N* = 7		
	Abdomen 2	41.75 ± 3.84	40.64 ± 3.36	38.67 ± 4.47	41.2 ± 3.80	38.12 ± 4.05		
	Supra-iliac	22.38 ± 2.87	22.83 ± 3.11	19.36 ±	24.4 ± 3.05	20.01 ± 2.97		
	Thigh	42.19 ± 2.80	41.19 ± 4.77	39.5 ±	43.88 ± 3.34	51.61 ± 1.50		
	Sum of 5 SKFs	155.9 ± 10.17	153.54 ± 12.02	145.55 ± 10.96	139.88 ± 11.57	142.03 ± 13.09		
	**Circumferences (cm)**							
	Subjects	*N* = 10	*N* = 10	*N* = 7	*N* = 7	*N* = 7		
	Abdominal	98.34 ± 3.60	95.26 ± 3.16	97.67 ± 3.65	95.21 ± 3.60	94.89 ± 4.46		
	Upper arm	29.25 ± 0.64	28.99 ± 0.68	29.24 ± 0.65	29.19 ± 0.65	29.00 ± 0.75		
	Calf	37.33 ± 0.90	38.28 ± 085	37.04 ± 0.65	37.74 ± 0.59	38.03 ± 0.70		
	Gluteal	106.94 ± 1.62	106.26 ± 1.72	106.69 ± 2.12	107.23 ± 2.41	106.8 ± 2.12		
	Thigh	61.11 ± 3.62	60.99 ± 1.76	63.44 ± 1.22	61.70 ± 1.24	59.93 ± 1.8		
	Sum of 7 Circs.	445.93 ± 8.49	443.16 ± 8.07	445.31 ± 7.40	440.51 ± 9.32	441.36 ± 8.56		
	W/H at umbilicus	0.92 ± 0.03	0.90 ± 0.03	0.91 ± 0.02	0.90 ± 0.03	0.89 ± 0.04		

**Table 7:** Dietary intake as a function of 15-week and 30-week training.

Variable	15-Week Slow Walkers	15-Week Fast Walkers			30-Week Slow Walkers	30-Week Fast Walkers		
Subjects	*N* = 11	*N* = 6	t_(df=14)_	*p*	*N* = 9	*N* = 7	t_(df=14)_	*p*
**Baseline intakes**								
Total calories (kcal)	2036.3 ± 111.2	1664.3 ± 115.9	2.10	0.053	1992.2 ± 179.5	1646.2 ± 229.5	1.21	0.281
Total carbohydrate (g)	252.2 ± 21.1	210.6 ± 17.6	1.30	0.213	240.9 ± 51.6	207.5 ± 34.2	0.50	0.641
Total protein (g)	84.7 ± 8.6	80.8 ± 9.6	0.28	0.782	72.5 ± 5.21	94.9 ± 13.4	1.75	0.141
Total Fat (g)	73.5 ± 7. 6	61.3 ± 5.1	1.10	0.289	66.0 ± 10.3	67.3 ± 8.3	0.09	0.928
**15-week intakes**								
Total calories (kcal)	1718.9 ± 139.0	1847.4 ± 122.3	0.60	0.556	1767.1 ± 217.1	2119.3 ± 41.0	1.36	0.232
Total carbohydrate (g)	250.5 ± 32.1	250.5 ± 24.3	0.05	1.0	233.0 ± 57.4	294.1 ± 14.5	0.89	0.416
Total protein (g)	67.7 ± 5.8	77.3 ± 7.3	0.99	0.338	74.2 ± 9.0	87.5 ± 8.4	1.05	0.343
Total fat (g)	51.75 ± 4.55	58.99 ± 3.81	1.05	0.3112	61.60 ± 2.19	64.21 ± 5.78	0.47	0.6551
**30-week intakes**								
Total calories (kcal)					1632.0 ± 142.6	2022.1 ± 549.0	0.80	0.461
Total carbohydrate (g)					239.7 ± 239.7	223.5 ± 43.31	0.34	0.747
Total protein (g)					59.73 ± 7.27	100.7 ± 29.5	1.57	0.178
Total fat (g)					63.85 ± 6.92	72.2 ± 15.3	0.55	0.606

## Data Availability

The data are available from the first author.

## References

[1] Fryar C.D., Carroll M.D., Afful J. (2021). Prevalence of Overweight, Obesity, and Severe Obesity among Adults Aged 20 and over: United States, 1960–1962 through 2017–2018. Vital Health Stat..

[2] Stierman B., Afful J., Carroll M.D., Chen T.-C., Davy O., Fink S., Fryar C.D., Gu Q., Hales C.M., Hughes J.P. (2021). National Health and Nutrition Examination Survey 2017–March 2020 prepandemic data files—Development of files and prevalence estimates for selected health outcomes. Natl. Health Stat. Rep..

[3] Hajer G.R., van Haeften T.W., Visseren F.L.J. (2008). Adipose tissue dysfunction in obesity, diabetes, and vascular diseases. Eur. Heart J..

[4] Murugan A., Sharma G. (2008). Obesity and respiratory diseases. Chron. Respir. Dis..

[5] Zuoi Q., Band S., Kesavados M., Erdogan Z.M. (2021). Obesity and postmenopausal hormone receptor-positive brest cancer: Epidemiology and mechanisms. Endocrinology.

[6] Calle E.E., Teras L.R., Thun M.J. (2005). Obesity and mortality. N. Engl. J. Med..

[7] Global BMI Mortality Collaboration (2016). Body-mass index and all-cause mortality: Individual-participant-data meta-analysis of 239 prospective studies in four continents. Lancet.

[8] Manson J.E., Willett W.C., Stampfer M.J., Colditz G.A., Hunter D.J., Hankinson S.E., Hennekens C.H., Speizer F.E. (1995). Body weight and mortality among women. N. Engl. J. Med..

[9] Vinneau J.M., Huibregtse B.M., Laidley T.M., Goode J.A., Boardman J.D. (2021). Mortality and obesity among U.S. older adults: The role of polygenic risk. J. Gerontol. B Psychol. Sci. Soc. Sci..

[10] Dwyer-Lindgren L., Freedman G., Engell R.E., Fleming T.D., Lim S.S., Murray C.J.L., Mokdad A.H. (2013). Prevalence of physical activity and obesity in US counties, 2001–2011: A road map for action. Popul. Health Metr..

[11] Hall K.D., Guo J., Dore M., Chow C.C. (2009). The progressive increase of food waste in America and its environmental impact. PLoS ONE.

[12] Arner P. (1997). Obesity and the adipocyte: Regional adiposity in man. J. Endocrinol..

[13] Astrup A. (1999). Physical activity and weight gain and fat distribution changes with menopause: Current evidence and research issues. Med. Sci. Sports Exerc..

[14] Franklin R.M., Ploutz-Snyder L., Kanaley J.A. (2009). Longitudinal changes in abdominal fat distribution with menopause. Metabolism.

[15] Sowers M., Zheng H., Tomey K., Karvonen-Gutierrez C., Jannausch M., Li X., Yosef M., Symons J. (2007). Changes in body composition in women over six years at midlife: Ovarian and chronological aging. J. Clin. Endocrinol. Metab..

[16] Folsom A.R., Kaye S.A., Thomas A.S., Hong C.P., Cerhan J.R., Potter J.D., Prineas R.J. (1993). Body fat distribution and 5-year risk of death in older women. JAMA.

[17] Lapidus F., Bengtsson C., Larsson B., Pennert K., Rybo E., Sjostrom L. (1984). Distribution of adipose tissue and risk of cardiovascular disease and death: A 12 year follow up of participants in the population study of women in Gothenburg, Sweden. BMJ.

[18] Pouliot M.C., Despres J.P., Lemieux S., Moorjani S., Bouchard C., Tremblay A., Nadeau A., Lupien P.J. (1994). Waist circumference and abdominal sagittal diameter: Best simple anthropometric indexes of abdominal visceral adipose tissue accumulation and related cardiovascular risk in men and women. Am. J. Cardiol..

[19] Schneider H.J., Glaesmer H., Klotsche J., Bohler H., Lehnert H., Zeiher A.M., Marz W., Pittrow D., Stalla G.K., Wittchen H.U. (2007). Accuracy of anthropometric indicators of obesity to predict cardiovascular risk. J. Clin. Endocrinol. Metab..

[20] Borer K.T., Fogleman K., Gross M., La New J., Dengel D. (2007). Walking intensity for postmenopausal bone mineral preservation and accrual. Bone.

[21] Borer K.T., Zheng Q., Jafari A., Javadi S., Kernozek T. (2019). Nutrient intake prior to exercise is necessary for increased osteogenic marker response in diabetic postmenopausal women. Nutrients.

[22] Zheng Q., Kernozek T., Daoud-Grey A., Borer K.T. (2021). Anabolic bone stimulus requires a pre-exercise meal and 45-minute walking impulse of suprathreshold speed-enhanced load and momentum to prevent or mitigate postmenopausal osteoporosis within circadian constraints. Nutrients.

[23] Houmard J.A., Tanner C.J., Slentz C.A., Duscha B.D., McCartney J.S., Kraus W.E. (2004). Effect of the volume and intensity of exercise training on insulin sensitivity. J. Appl. Physiol..

[24] Ballor D.L., Keesey R.E. (1991). A meta-analysis of the factors affecting exercise-induced changes in body mass, fat mass and fat-free mass in males and females. Int. J. Obes..

[25] Chin S.H., Kahathuduwa C.N., Binks M. (2016). Physical activity and obesity: What we know and what we need to know. Obes. Rev..

[26] Jakicic J.M., Rogers R.J., Davis K.K., Collins K.A. (2018). Role of physical activity and exercise in treating patients with overweight and obesity. Clin. Chem..

[27] Ready A.E., Naimark B., Ducas J., Sawatzky J.V., Boreskie S.L., Drinkwater D.T., Oosterveen S. (1996). Influence of walking volume on health benefits in women post-menopause. Med. Sci. Sports Exerc..

[28] Donnelly J.E., Honas J.J., Smith B.K., Mayo M.S., Gibson C.A., Sullivan D.K., Lee J., Herrmann S.D., Lambourne K., Washburn R.A. (2013). Aerobic exercise alone results in clinically significant weight loss for men and women: Midwest exercise trial 2. Obesity.

[29] Ballor D.L., McCarthy J.P., Wilterdink E.J. (1990). Exercise intensity does not affect the composition of diet- and exercise-induced body mass loss. Am. J. Clin. Nutr..

[30] Gaesser G.A., Rich R.G. (1984). Effects of high-and low-intensity exercise training on aerobic capacity and blood lipids. Med. Sci. Sports Exerc..

[31] Grediagin M.A., Cody M., Rupp J., Benardot D., Shern R. (1995). Exercise intensity does not affect body composition change in untrained, moderately overfat women. J. Am. Diet. Assoc..

[32] Duncan J.J., Gordon N.F., Scott C.B. (1991). Women walking for health and fitness. How much is enough?. JAMA.

[33] Irving B.A., Davis C.K., Brock D.W., Weltman J.Y., Swift D., Barrett E.J., Gaesser G.A., Weltman A. (2008). Effect of exercise training intensity on abdominal visceral fat and body composition. Med. Sci. Sports Exerc..

[34] Mezghanni N., Chaabouni K., Chtourou H., Masmoudi L., Chamari K., Lasoued A., Mnif M., Jamoussi K., Mejdoub H. (2012). Effect of exercise training intensity on body composition, lipid profile, and insulin resistance in young obese women. Afr. J. Microbiol. Res..

[35] Girandola R.N. (1976). Body composition changes in women: Effect of high and low exercise intensity. Arch. Phys. Med. Rehabil..

[36] Van Aggel-Leijssen D.P., Saris W.H., Homan M., van Baak M.A. (2001). The effect of exercise training on beta-adrenergic stimulation of fat metabolism in obese men. Int. J. Obes. Relat. Metab. Disord..

[37] Miyatake N., Nishikawa H., Morishita A., Kunitomni M., Wada J., Suzuki H., Takahashi K., Makino H., Kira S., Fuji M. (2002). Daily walking reduces visceral adipose tissue areas and improves insulin resistance in Japanese obese subjects. Diabetes Res. Clin. Pract..

[38] Tremblay A., Simoneau J.A., Bouchard C. (1994). Impact of exercise intensity on body fatness and skeletal muscle metabolism. Metabolism.

[39] Abe T., Kawakami Y., Sugita M., Fukunaga T. (1997). Relationship between training frequency and subcutaneous and visceral fat in women. Med. Sci. Sports Exerc..

[40] Rwin M.L., Yasui Y., Ulrich C.M., Bowen D., Rudolph R.E., Schwartz R.S., Yukawa M., Aiello E., Potter J.D., McTiernan A. (2003). Effect of exercise on total and intra-abdominal body fat in postmenopausal women: A randomized controlled trial. JAMA.

[41] Slentz C.A., Duscha B.D., Johnson J.L., Ketchum K., Aiken L.B., Samsa G.P., Houmard J.A., Bales C.W., Kraus W.E. (2004). Effects of the amount of exercise on body weight, body composition, and measures of central obesity: STRRIDE-a randomized controlled study. Arch. Intern. Med..

[42] Slentz C.A., Aiken L.B., Houmard J.A., Bales C.W., Johnson J.L., Tanner C.J., Duscha B.D., Kraus W.E. (2005). Inactivity, exercise, and visceral fat. STRRIDE: A randomized controlled study of exercise intensity and amount. J. Appl. Physiol..

[43] Romijn J.A., Coyle E.F., Sidosis L.S., Gastaldelli A., Horowitz J.F., Endert E., Wolfe R.R. (1993). Regulation of endogenous fat and carbohydrate metabolism in relation to exercise intensity and duration. Am. J. Physiol. Endocrinol. Metab..

[44] Cavagna G.A., Margaria R. (1966). Mechanics of walking. J. Appl. Physiol..

[45] Reybrouck T., Ghesquiere J., Weymans M., Amery A. (1986). Ventilatory threshold measurement to evaluate maximal endurance performance. Int. J. Sports Med..

[46] Davis J.A., Vodak P., Wilmore J.H., Vodak J., Kurtz P. (1976). Anaerobic threshold and maximal aerobic power for three modes of exercise. J. Appl. Physiol..

[47] Heyward V.H., Solarczyk L.M. (1996). Applied Body Composition Assessment.

[48] Lohman T.G., Roche A.F., Martorell R. (1988). Anthropometric Standardization Reference Manual.

[49] Teran J.C., Sparks K.E., Quinn L.M., Fernandez B.S., Krey S.H., Steffee W.P. (1991). Percent body fat in obese white females predicted by anthropometric measurements. Am. J. Clin. Nutr..

[50] Jackson A.S., Pollock M.L., Ward A. (1980). Generalized equations for predicting body density of women. Med. Sci. Sports Exerc..

[51] Siri W., Brozek J., Henschel A. (1961). Body composition from fluid spaces and density: Analysis of methods. Techniques for Measuring Body Composition.

[52] Murray M.P., Drought A.B., Kory R.C. (1964). Walking patterns of normal men. J. Bone Jt. Surg..

[53] Münkler P., Kröger S., Liosis S., Abdin A., Lyan E., Eitel C., Eitel I., Meyer C., Willems S., Heeger C.-H. (2018). Ablation index for catheter ablation of atrial fibrillation—Clinical applicability and comparison with force-time integral. Circ. J..

[54] Greendale G.A., Han W., Finkelstein J.S., Burnett-Bowie S.-A.M., Huang M.H., Marin D., Karlamangla A.S. (2021). Changes in regional fat distribution and anthropometric measures across the menopause transition. J. Endocrinol. Metab..

[55] Franklin B., Buskirk E., Hodgson J., Gahagan H., Kollias J., Mendez J. (1979). Effects of physical conditioning on cardiorespiratory function, body composition and serum lipids in relatively normal-weight and obese middle-aged women. Int. J. Obes..

[56] Epstein L.H., Wing R.R. (1980). Aerobic exercise and weight. Addict. Behav..

[57] Leon A.S., Conrad J., Hunninghake D.B., RSerfass R. (1979). Effects of a vigorous walking program on body composition, and carbohydrate and lipid metabolism of obese young men. Am. J. Clin. Nutr..

[58] Coggan A.R., Kohrt W.M., Spina R.J., Bier D.M., Holloszy J.O. (1990). Endurance training decreases plasma glucose turnover and oxidation during moderate-intensity exercise in men. J. Appl. Physiol..

[59] Coggan A.R., Raguso C.A., Gastaldelli A., Sidossis L.S., Yeckel C.W. (2000). Fat metabolism during high-intensity exercise in endurance-trained and untrained men. Metab. Clin. Exper..

[60] Coggan A.R., Raguso C.A., Williams B.D., Sidossis L.S., Gastaldelli A. (1995). Glucose kinetics during high-intensity exercise in endurance-trained and untrained humans. J. Appl. Physiol..

[61] McMurray R.G., Ben-Ezra V., Forsythe W.A., Smith A.T. (1985). Responses of endurance-trained subjects to caloric deficits induced by diet or exercise. Med. Sci. Sports Exerc..

[62] Kohrt W.M., Ehsani A.A., Birge S.J. (1997). Effects of exercise involving predominantly either joint-reaction or ground-reaction forces on bone mineral density in older women. J. Bone Miner. Res..

[63] Perrini S., Leonardini A., Laviola L., Giorgino F. (2008). Biological specificity of visceral adipose tissue and therapeutic intervention. Arch. Physiol. Biochem..

[64] Moody D.L., Kollias J., Buskirk E.R. (1969). The effect of moderate exercise program on body weight and skinfold thickness in overweight college women. Med. Sci. Sports.

